# Health Benefits, Risks, and Cost-Effectiveness of Influenza Vaccination of Children

**DOI:** 10.3201/eid1210.051015

**Published:** 2006-10

**Authors:** Lisa A. Prosser, Carolyn Buxton Bridges, Timothy M. Uyeki, Virginia L. Hinrichsen, Martin I. Meltzer, Noelle-Angelique M. Molinari, Benjamin Schwartz, William W. Thompson, Keiji Fukuda, Tracy A. Lieu

**Affiliations:** * Harvard Medical School, Boston, Massachusetts, USA;; † Harvard Pilgrim Health Care, Boston, Massachusetts, USA;; ‡ Centers for Disease Control and Prevention, Atlanta, Georgia, USA;; § Children's Hospital, Boston, Massachusetts, USA

**Keywords:** influenza, vaccination, child health, cost-effectiveness analysis, economic, dispatch

## Abstract

We estimated cost-effectiveness of annually vaccinating children not at high risk with inactivated influenza vaccine (IIV) to range from US $12,000 per quality-adjusted life year (QALY) saved for children ages 6–23 months to $119,000 per QALY saved for children ages 12–17 years. For children at high risk (preexisting medical conditions) ages 6–35 months, vaccination with IIV was cost saving. For children at high risk ages 3–17 years, vaccination cost $1,000–$10,000 per QALY. Among children not at high risk ages 5–17 years, live, attenuated influenza vaccine had a similar cost-effectiveness as IIV. Risk status was more important than age in determining the economic effects of annual vaccination, and vaccination was less cost-effective as the child's age increased. Thus, routine vaccination of all children is likely less cost-effective than vaccination of all children ages 6–23 months plus all other children at high risk.

## Abstract 2

Vaccinating children aged 6–23 months, plus all other children at high-risk, will likely be more effective than vaccinating all children against influenza.

The risks of influenza, both annual epidemic and pandemic, have caused national policymakers to ask whether routine influenza vaccination should be expanded to healthy children and adults of all ages. During the 2003–04 influenza season, reports of >150 influenza-associated deaths among children and unprecedented demand for influenza vaccine highlighted the need to reevaluate the nation's influenza vaccination policies regarding children (*1**–**3*). The Advisory Committee on Immunization Practices (ACIP) and the American Academy of Pediatrics Red Book Committee have recommended that all children 6–23 months of age and their household contacts should receive annual influenza vaccination, and this policy has been widely adopted (*4**,**5*). In February 2006, the ACIP recommended expanding routine influenza vaccination to children 24–59 months old (L. Pickering, pers. comm.). However, a vote to recommend routine influenza vaccination for all children and adults failed. ACIP members requested more information on the projected health benefits, cost-effectiveness, and logistical issues regarding expanding influenza recommendations to other age groups.

Should influenza vaccine be routinely used in older children without high-risk conditions? This question is especially relevant, given the introduction of live, attenuated (intranasal) influenza vaccine (LAIV) for healthy persons ages 5–49 years, which has a higher list price than the inactivated (injected) vaccine but is also potentially more effective (*6**,**7*). Previous studies have examined the cost-effectiveness of influenza vaccination in various age groups (*8**–**10*). However, these studies may have been overly optimistic regarding vaccination because they assumed high influenza attack rates, low estimates for vaccination costs, or both, thereby limiting their use in policy decisions. Further, no studies have been published that compare the cost-effectiveness of live attenuated influenza vaccines with that of inactivated influenza vaccines.

Our objective in this study was to evaluate the cost-effectiveness of routine annual influenza vaccination, comparing live attenuated with inactivated vaccines, for children in varying age and risk groups from 6 months to 17 years. This is the first study to include measures of health preferences that allow results to be calculated in quality-adjusted life years (QALYs).

## Methods

**Figure 1:**
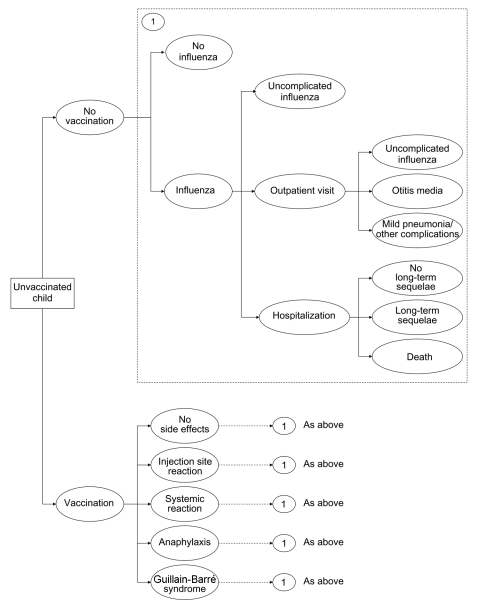
Influenza cost-effectiveness model. Each health state in the model is associated with a cost and quality adjustment from Table 1.

Using standard software (TreeAge Pro 2004 Software, release 6, Treeage Software, Williamstown, MA, USA), we created a mathematical model (decision tree) to estimate the effect of influenza vaccination on outcomes and costs among children. The decision tree evaluated 3 options: 1) no vaccination; 2) inactivated influenza vaccine (IIV); and 3) live, attenuated influenza vaccine (LAIV). It estimated costs and outcomes for influenza-related illness for children stratified into 10 subgroups by age (6–23 months, 2 years [24–35 months], 3–4 years, 5–11 years, 12–17 years) and risk status (high risk or not at high risk). Children were defined as being at high risk for influenza-related complications due to preexisting medical conditions (*4*). Since most costs and consequences related to influenza occur during a single influenza season, the time horizon of the decision tree was 1 year. Costs and effects of long-term outcomes (death, long-term sequelae of influenza-related hospitalization or vaccine adverse events), however, were also included in the model. A simplified schematic of the decision tree is shown in Figure 1. Input parameters for probabilities, costs, and outcomes were described by using probability distributions (Table 1, Table 2, and Table 3).

**Table 1:** Model inputs and assumptions for children ages 6 months to 17 years*†

Variable			Most likely estimate	Range for sensitivity analysis
Influenza illness attack rate (annual)				
	6-23 mo		0.157	0.02–0.35
	2 y		0.155	0.02–0.35
	3–4 y		0.155	0.01–0.35
	5–11 y		0.08	0.01–0.18
	12–17 y		0.06	0.01–0.14
Probability of outpatient visit for child with influenza illness‡				
	6–23 mo		0.5	0.17–0.83
	2 y		0.47	0.15–0.81
	3–4 y		0.43	0.12–0.78
	5–11 y		0.28	0.11–0.5
	12–17 y		0.24	0.06–0.5
Probability of otitis media for child with medically attended influenza illness				
	6–23 months		0.63	0.33–0.8
	2 y		0.58	0.27–0.8
	3–4 y		0.39	0.17–0.6
	5–11 y		0.23	0.05–0.5
	12–17 y		0.15	0.01–0.4
Probability of nonhospitalized pneumonia or other outpatient complication for child with medically attended influenza illness§				
	6–23 mo		0.2	0.04–0.5
	2 y		0.15	0.02–0.4
	3–4 y		0.15	0.02–0.4
	5–11 y		0.11	0.02–0.3
	12–17 y		0.08	0.01–0.2
Hospitalizations for pneumonia or other respiratory conditions due to influenza/10,000 children not at high risk¶				
	6-23 mo		28.3	1.9–80.0
	2 y		17.1	0–56.8
	3–4 y		8.0	0–35.4
	5–11 y		3.1	0–16.0
	12–17 y		3.1	0–14.9
Probability of long-term sequelae following influenza-related hospitalization‡			0.01	0.001–0.03
Probability of death during influenza-related hospitalization			0.0009	0–0.002
Vaccine effectiveness in preventing influenza illness#				
	IIV		0.69	0.4–0.9
	LAIV		0.838	0.6–0.96
Probability of medically attended vaccination-related adverse events				
	Injection site reaction			
		6-23 mo	0.008	0.002–0.017
		2 y	0.003	0.001–0.006
		3–4 y	0.002	0.0004–0.003
		5–11 y	0.001	0.0002–0.002
		12–17 y	0.0003	0.0001–0.001
	Systemic reaction (fever)**			
		6–23 mo	0.013	0.001–0.025
		2 y	0.011	0.0008–0.020
		3–4 y	0.009	0.0007–0.016
		5–11 y	0.004	0.0003–0.008
		12–17 y	0.003	0.0002–0.005
	Anaphylaxis		0.00000025	0–0.000001
	Guillain-Barré syndrome		0.000001	0–0.00001

*IIV, inactivated influenza vaccine; LAIV, live, attenuated influenza vaccine. †Refer to Table A1 for list of references used to derive model inputs. ‡Estimates for children not at high risk are shown. Probabilities are estimated to be twice as high for children at high risk for influenza-related complications. §Estimates for healthy children shown. Probabilities are estimated to be <5 times as high for children at high risk for influenza-related complications. Most likely estimates for children at high risk are 1.6 times as high as for healthy children. ¶Children at high-risk are estimated to be hospitalized at 3–6 times the rate of children not at high risk. #Assumes vaccine is poorly matched with circulating virus 1 in 10 years (i.e., vaccine effectiveness is assumed to be 0 years with a poor match). **Definitions and follow-up for incidence of fever following vaccination vary by study. Rates are 2× higher for children at high risk.

**Table 2:** US $ cost inputs for children ages 6 months to 17 years*

Cost input		Most likely estimate	Range for sensitivity analysis
Influenza-related costs			
	OTC medications†	$3	
	Physician visit, uncomplicated influenza‡	$27	$10–$78
	Physician visit, otitis media§	$78	$23–$197
	Physician visit, non-hospitalized pneumonia§	$179	$62–$715
	Hospitalization¶	$4,300	$1,300–$34,500
	Long-term sequelae following influenza-related hospitalization#	$625,000	$0–$1,000,000
Vaccination costs			
	Per dose, IIV** (children <3 y)	$9.56††	1×–4× base case
	Per dose, IIV** (children >3 y)	$6.86††	
	Per dose, LAIV**	$12.89††	$10–$25
	Administration costs (0–2 visits)¶¶	$25	$10–$40
	Parent time costs, per visit##	$32	$0–$64
	Total vaccination costs***		$30–$110
	6-23 mo	$79	
	2 y	$66	
	3–4 y	$59	
	5–11 y	$49	
	12-17 y	$49	
Vaccination-related adverse events			
	Physician visit for injection site reaction†††	$61	$30–$683
	Anaphylaxis‡‡‡	$2,700	$52–$13,754
	Guillain-Barré syndrome§§§	$23,360	$6,700–$78,900

*OTC, over the counter; IIV, inactivated influenza vaccine; LAIV, live, attenuated influenza vaccine. †Vary by age, calculated by costing out recommended dose of acetaminophen for average weight in each age group. ‡Only a proportion of children with influenza illness are assumed to make a physician visit. ICD-9 codes: 487 and 487.0. §Costs of physician visits for otitis media and nonhospitalized pneumonia vary by age group and include prescription medications and laboratory tests. Costs shown are for children 6–23 mo. See Appendix for full list of costs by age. ¶ICD-9 codes: 460-466, 471-474, 477, 478, 480-483, 490-496, 506-508, 510, 511, 514, 518, 519. #Includes costs of lifetime care and special education. **2 doses assumed for children <5 y receiving their first influenza vaccination. ††Vaccine dose costs are based on 2004 CDC-negotiated prices. Cost for children <3 y assumes thimerosal-free vaccine is used. ‡‡Current Procedural Terminology (CPT) codes: 99211 for an additional visit ($19.95) and 90471 for a vaccination at an existing visit ($10.37). §§Each physician visit is assumed to take 2 hours of parent time valued at an average hourly wage rate of $15.54. ¶¶Proportion of children requiring 2 doses is 1 for 6–23 mo, 0.5 for 2 y, and 0.33 for 3–4 y. No. of additional visits needed to administer recommended number of vaccine doses is 1.07 for 6–23 mo, 0.91 for 2 y, and 0.84 for 3–4 y, and 0.75 for 5–17 y. See Appendix for more details. Total vaccination costs in Table 1 exclude average costs for vaccination-related adverse events of $0.18–$2.05 per child, depending on age and risk status. ##5-minute visit, CPT code 99211. ***ICD-9 codes: 999.4, 995.0, 995.6x. †††ICD-9 code: 357.0.

**Table 3:** Quality adjustments for influenza-related illness and vaccination-related adverse events (decrease in utility)*†

Events	Most likely estimate	Range for sensitivity analysis
Episode of influenza	0.005	0.002-0.009
Otitis media	0.042	0.023-0.065
Nonhospitalized complications (pneumonia)	0.046	0.027-0.071
Hospitalization, pneumonia	0.076	0.054-0.100
Anaphylaxis	0.020	0.006-0.041
Guillain-Barré syndrome	0.141	0.092-0.199

*Quality adjustments are included in model as a one-time decrement in utility for each temporary health state. For example, an episode of influenza results in a 1-time loss of 0.005 quality-adjusted life years (QALYs). Utility losses were calculated by dividing discounted time-traded off by respondent's discounted life expectancy. See appendix for references. †Average life span used to calculate total QALYs lost due to lifelong sequelae and death was 77.9–78.2 years, depending on child's current age.

### Natural History of Influenza

Influenza-related outcomes included in the decision tree were episodes of influenza illness (medically attended or not), otitis media, mild pneumonia (and other complications treated on an outpatient basis), hospitalizations (with and without long term sequelae), and deaths. Event rates, by age and risk group, were derived from the published literature and were supplemented by expert opinion where data were limited or unavailable (Table 1) (11–15) (Appendix)

### Vaccine Effectiveness

Inactivated vaccine was considered for all 10 subgroups, and LAIV was considered only for children not at high risk. Children 6 months to 4 years were included as a theoretical intended population for LAIV, although LAIV is currently licensed in the United States only for children 5–17 years. The most likely estimate for vaccine effectiveness against symptomatic influenza illness was lower for IIV (0.690) than the most likely estimate for LAIV (0.838) (Table 1) (*16**,**17*).

### Vaccination-related Adverse Events

Adverse events attributable to influenza vaccination included in the decision tree were medically-attended episodes of injection site reactions, systemic reactions (defined as fever within 2–7 days of vaccination), anaphylaxis, and Guillain-Barré syndrome (Table 1). Probabilities of medically-attended vaccine adverse events were highest for the youngest age group and declined as age increased.

### Costs

Costs included direct medical costs (physician visits, over-the-counter remedies, prescription drugs, diagnostic tests, and hospitalizations) and opportunity costs (parent time costs) for physician visits (Table 2). All costs were adjusted to 2003 dollars by using the medical cost component of the Consumer Price Index (available from http://www.bls.gov/cpi/). Costs of physician visits for influenza illness, influenza-related hospitalizations, and vaccination-related adverse events were calculated by using a large database that reported payments for health insurance companies in the mid-Atlantic states of the United States (The Medstat Group, Ann Arbor, MI, USA). Vaccination costs included vaccine dose costs, administration costs, medical attention for vaccine adverse events, and, if an additional visit was required, parent time costs (*18**,**19*).

It is recommended that first-time recipients aged 6 months through 8 years receive 2 doses of influenza vaccine (*4*). Some children will also require additional office visits to be vaccinated with either 1 or 2 doses. The mean number of additional office visits needed to deliver the recommended number of doses ranged from 1.07 for children ages 6–23 months to 0.75 for children ages 5–17 years (Table 2) (*20*).

### Health Outcomes

The model projected 4 different outcomes that were averted through vaccination: influenza episodes, hospitalizations, deaths, and QALYs. The QALY is a measure of net health effects that takes into account the health benefits of averted influenza cases as well as the health costs of vaccination-related adverse events. We obtained QALY valuations for each health event in the model from 2 studies (Table 3) (*21**,**22*). In these studies, adult respondents were asked for the amount of time that they were willing to give up from the end of their life to prevent a specific temporary health state in a hypothetical child. We explicitly asked respondents to include a parent's reduction in quality of life associated with a child's illness and any time lost from work to care for a sick child in the time-tradeoff valuation; therefore, time-tradeoff amounts could exceed the length of the event. QALYs lost due to severely disabling long-term sequelae after influenza hospitalization, such as acute necrotizing encephalopathy with irreversible neurologic damage, were also included (*23**,**24*). An influenza-related death was assumed to result in the loss of 1 QALY for each year of life lost.

### Analysis Plan

**Figure A1:**
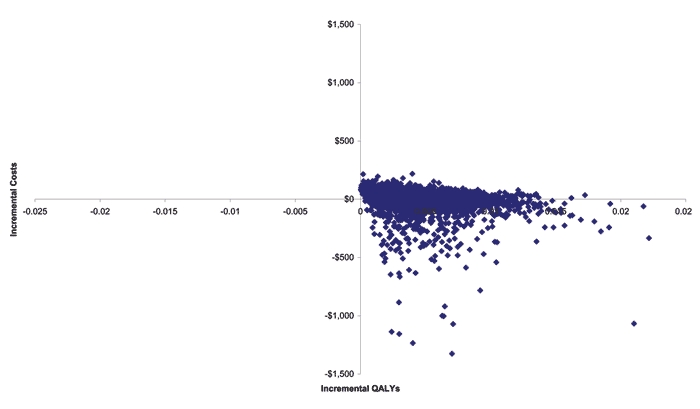
Scatterplot of incremental costs and quality-adjusted life years (QALYs) for children ages 6–23 months not at high risk from probabilistic sensitivity analysis.

The primary outcome measure was the incremental cost-effectiveness ratio in dollars per QALY. Secondary measures included costs and events averted per 1,000 vaccinated children, dollars per influenza-related event avoided, dollars per hospitalization avoided, and dollars per death averted. One-way sensitivity analyses were conducted on all variables, in which the impact on the average $/QALY saved was examined by altering each variable within the range of given values (Table 1). Two-way sensitivity analyses were conducted on variables for which the results were most sensitive in 1-way sensitivity analysis. A scenario analysis examined the effect of excluding parent time costs. Another scenario analysis evaluated the effect of using an alternative calculation for quality adjustments, which used the duration of the health state in the child as the denominator instead of respondent's life expectancy. To evaluate the effects of parameter uncertainty, a probabilistic sensitivity analysis was conducted. For the probabilistic sensitivity analysis, each variable was assigned a distribution of possible values, assuming β distributions for probabilities and quality adjustments and log-normal distributions for costs (Appendix, Figure A1). For each run in the probabilistic sensitivity analysis, the model randomly picked a different value for each variable from its associated distribution. The model was run 10,000 times for each age-risk and vaccine combination separately. Cost-effectiveness acceptability curves show the cumulative probabilities of the cost-effectiveness ratio, from $0 to $250,000/QALY, due to vaccinating children against influenza (i.e., the curves display the probability of the cost-effectiveness being less than or equal to a given $/QALY amount), by using the results from the Monte Carlo analysis.

## Results

### Health Benefits, Risks, and Costs

Influenza vaccination with IIV was projected to be cost saving for children ages 6–35 months at high risk and to require a net investment for all other age and risk groups. The projected benefits of vaccination decreased as age increased (Table 4). For example, routine influenza vaccination with IIV of children 6–23 months old not at high risk was projected to avert 108 influenza events per 1,000, while vaccination of 5- to 11-year-old children was projected to averted 55 influenza events per 1,000. Among the 5- to 11-year-olds not at high risk, the projected number of influenza-related hospitalizations and deaths averted by influenza vaccination with IIV was only one tenth the number averted among 6- to 23-month-old children not at high risk. For children not at high-risk age >5 years, the number of projected influenza events averted was similar for LAIV and IIV.

**Table 4:** Health benefits, risks, and costs of influenza vaccination of varying age and risk groups per 1,000 children vaccinated, means* (95% CI†)

			Net costs, $‡	Influenza events averted (all)	Influenza hospitalizations averted	Deaths averted	Vaccine adverse events incurred§	QALYs gained
Using inactivated influenza vaccine								
	Non-high risk							
		6–23 mo	37,000 (–119,000 to 98,000)	108 (16–276)	2 (0.2–6)	0.002 (0–0.007)	21 (8–47)	3.0 (0.4–9.0)
		2 y	43,000 (–40,000 to 83,000)	107 (15–276)	1.2 (0.1–4.2)	0.001 (0–0.005)	14 (5–30)	2.4 (0.3–7.3)
		3–4 y	47,000 (2,000–78,000)	107 (15–276)	0.6 (0–2.3)	0.0005 (0–0.0025)	10 (3–24)	1.7 (0.2–5.2)
		5–11 y	44,000 (21,000–68,000)	55 (8–142)	0.2 (0–0.7)	0.0002 (0– 0.0008)	5 (2–11)	0.6 (0.1–1.7)
		12–17 y	44,000 (22,000– 68,000)	41 (6–104)	0.2 (0–0.6)	0.0002 (0–0.0008)	3 (1–8)	0.4 (0–1.1)
	High risk							
		6–23 mo	–74,000) (–552,000 to 83,000)	108 (16–276)	5.5 (0.5–6.5)	0.005 (0–0.020)	32 (11–56)	7.2 (0.8–23.2)
		2 y	–22,000) (–292,000 to 72,000)	107 (15–276)	3.5 (0.2–11.4)	0.003 (0–0.013)	25 (7–44)	5.4 (0.6–17.2)
		3–4 y	2,000 (–212,000 to 70,000)	107 (15–276)	2.2 (0.1–9.1)	0.002 (0–0.010)	19 (5–37)	4.0 (0.4–13.1)
		5–11 y	12,000 (–125,000 to 59,000)	55 (8–142)	1.3 (0.1–3.9)	0.001 (0–0.005)	9 (3–24)	1.6 (0.2–5.6)
		12–17 y	13,000 (–120,000 to 59,000)	41 (6–104)	1.3 (0.1–3.9)	0.001 (0–0.005)	6 (1–15)	1.3 (0.1–4.5)
Using LAIV¶								
	Non-high risk							
		*6–23 mo*	*32,000 (–155,000 to 99,000)*	*132 (20–319)*	*2.4 (0.3–7.2)*	*0.002 (0–0.009)*	13 (3–32)	*3.7 (0.5–10.5)*
		*2 y*	*42,000 (–59,000 to 85,000)*	*130 (20–322)*	*1.4 (0.1–4.9)*	*0.001 (0–0.005)*	11 (2–26)	*2.9 (0.4–8.5)*
		*3–4 y*	*50,000 (–3,000 to 83,000)*	*130 (20–322)*	*0.7 (0–2.7)*	*0.0006 (0–0.0029)*	9 (2–23)	*2.1 (0.3–6.1)*
		5–11 y	48,000 (22,000–73,000)	67 (10–166)	0.3 (0–0.8)	0.0002 (0–0.0010)	4 (1–10)	0.7 (0.1–1.9)
		12–17 y	49,000 (23,000–73,000)	50 (8–120)	0.3 (0–0.7)	0.0002 (0–0.0010)	3 (0–7)	0.5 (0.1–1.3)

*CI, confidence interval; QALYs, quality-adjusted life years; LAIV, live, attenuated influenza vaccine. †Bootstrapped. ‡Net costs = costs of vaccination minus savings from disease averted. §Includes medically attended injection site reactions, systemic reactions, anaphylaxis, and Guillain-Barré syndrome. ¶Italics indicate that LAIV is not licensed for children <5 y.

### QALYs and Cost-Effectiveness

All vaccination strategies had net positive QALYs gained, which indicated that the health benefits of vaccination outweighed the risks (Table 4). For children not at-high risk, the QALYs gained by IIV use were highest for 6- to 23-month-olds at 3.0 QALYs gained per 1,000 children vaccinated, compared with 2.4 per 1,000 children vaccinated for 2-year-olds and 1.7 per 1,000 children vaccinated for 3- to 4-year-olds. For children at high risk, the QALYs gained by IIV use ranged from 1.3 to 7.2 per 1,000 children vaccinated, depending on age group. For children 5–17 years old not at high risk, LAIV use would result in slightly higher QALYs gained because of the vaccine's higher effectiveness at 0.5 to 3.7 per 1,000 children vaccinated.

IIV use was cost saving among children at high risk ages 6 months to 2 years (Table 5). For children <5 years not at high risk as well as children at high risk in all age groups, IIV use had mean cost-effectiveness ratios of <$30,000 per QALY saved. Cost-effectiveness ratios based on dollars per influenza episode averted yielded patterns similar to the ratios of dollars per QALY saved, ranging from cost savings for children at high risk ages <2 years to $1,070 per influenza case averted for healthy 12- to 17-year-olds (Table 5).

**Table 5:** Incremental cost-effectiveness ratios for use of inactivated and live attenuated influenza vaccination in varying age and risk groups compared to no vaccination, mean (2.5% and 97.5% bootstrapped percentiles)*

Age group		Using inactivated influenza vaccine		Using live, attenuated influenza vaccine†
		Children not at high risk	Children at high risk	Children not at high risk
$ per influenza episode averted‡				
	6–23 mo	340 (CS–4,690)	CS (CS–4,090)	*240 (CS–3,890)*
	2 y	400 (CS–3,990)	CS (CS–3,620)	*330 (CS–3,340)*
	3–4 y	440 (10–3,590)	20 (CS–3,410)	*440 (CS–3,170)*
	5–11 y	800 (180–5,850)	210 (CS–5,560)	720 (170–5,290)
	12–17 y	1,070 (250–7,780)	310 (CS–7,360)	980 (240–7,070)
$ per hospitalization averted‡				
	6-23 mo	19,000 (CS–350,000)	CS (CS–132,000)	*14,000 (CS–287,000)*
	2 y	37,000 (CS–633,000)	CS (CS–232,000)	*30,000 (CS–522,000)*
	3–4 y	84,000 (1,000–2,587,000)	1,000 (CS–750,000)	74,000 (CS–2,227,000)
	5–11 y	202,000 (38,000–1,929,000)	9,000 (CS–310,000)	184,000 (35,000–1,629,000)
	12–17 y	206,000 (43,000–1,768,000)	10,000 (CS–304,000)	188,000 (40,000–1,575,000)
$ per death averted‡				
	6–23 mo	22 m (CS–1,109 m)	CS (CS–342 m)	*16 m (CS–880 m)*
	2 y	42 m (CS–1,762 m)	CS (CS–591 m)	*34 m (CS–1,435 m)*
	3–4 y	98 m (1 m–6,840 m)	1 m (CS–1,873 m)	*86 m (CS;5,991 m)*
	5–11 y	234 m (32 m–5,993 m)	10 m (CS–876 m)	212 m (32 m–5,331 m)
	12–17 y	238 m (37 m–5,607 m)	12 m (CS–892 m)	217 m (34 m–5,007 m)
$ per quality-adjusted life-year saved				
	6–23 mo	12,000 (CS–208,000)	CS (CS–85,000)	*9,000 (CS–167,000)*
	2 y	18,000 (CS–217,000)	CS (CS–100,000)	*15,000 (CS–180,000)*
	3–4 y	28,000 (1,000–290,000)	1,000 (CS–130,000)	*25,000 (CS–236,000)*
	5–11 y	79,000 (15,000–682,000)	7,000 (CS–260,000)	72,000 (14,000–592,000)
	12–17 y	119,000 (24,000–1,040,000)	10,000 (CS–367,000)	109,000 (22,000–888,000)

*CS, cost savings; m, million. †Numerator does not include productivity losses. ‡Italics indicate that live, attenuated influenza vaccine is not licensed for children <5 y.

Using base-case vaccine purchase prices for LAIV and IIV (Table 2), LAIV for children ages 5–17 years not at high risk had higher mean net costs and yielded greater mean health benefits than IIV. The cost-effectiveness ratios for LAIV were $72,000 per QALY gained for 5- to 11-year-olds and $109,000 per QALY gained for 12- to 17-year-olds (Table 5).

### Sensitivity Analyses

Probabilistic sensitivity analysis provided confidence intervals for projected costs and events averted and quasi-confidence intervals for cost-effectiveness ratios. By using base case assumptions, results for LAIV are slightly more favorable than IIV (compared to no vaccination), and vaccination with LAIV is the preferred strategy. However, probabilistic sensitivity analysis indicated projected results were similar for IIV and LAIV.

**Figure 2:**
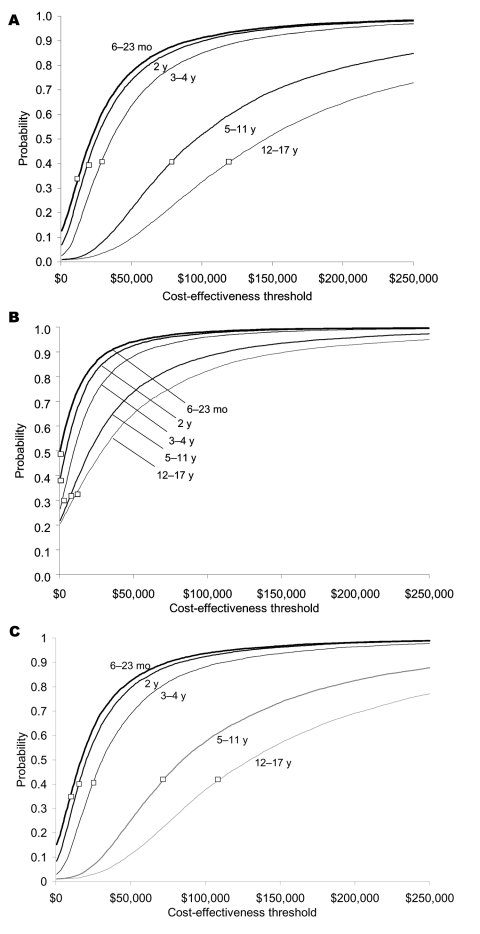
Cost-effectiveness acceptability curves for inactivated influenza vaccine compared with no vaccination (A, children not a high risk; B, children at high risk). Cost-effective acceptability curves for live, attenuated vaccine compared with no vaccine (C, children not at high risk only). Box indicates the mean cost-effectiveness ratio.

Cost-effectiveness acceptability curves generated through probabilistic sensitivity analysis are very similar for IIV and LAIV (Figure 2A and Figure 2C). The probability that the cost-effectiveness of IIV would be <$30,000/QALY ranged from 51% to 89% for all children ages 6–23 months and 2 years (Figure 2). For children of any age not at high risk, the probability that IIV would be cost saving was <10% (Figure 2A). For children aged >5 years not at high risk, the probability that the cost-effectiveness of LAIV, compared with no vaccination, would be <$30,000 per QALY gained was 5%–13% (Figure 2C).

**Figure 3:**
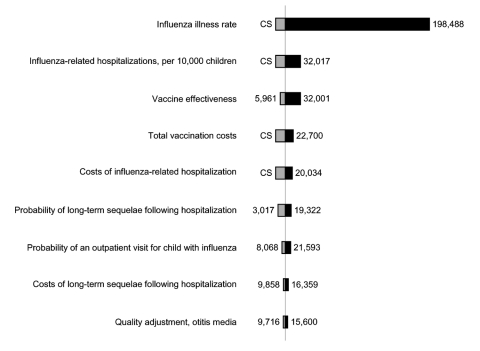
One-way sensitivity analyses on selected variables for children ages 6–23 months not at high risk, in dollars per quality-adjusted life years gained. This figure reports variables to which the results were most sensitive. Variables not reported here had less effect on results than those included above. Base case=$12,300.

In 1-way sensitivity analyses, cost-effectiveness ratios were most sensitive to changes in influenza illness attack rate, hospitalization rates, total vaccination costs, and vaccine effectiveness (Figure 3). Cost-effectiveness ratios varied notably with total costs of vaccination for IIV. For example, if total costs of vaccination were doubled for children ages 6–23 months who were not at high risk, cost-effectiveness ratios increased (worsened) by a factor of 3. We included costs for parent time associated with taking a child to the physician's office to receive influenza vaccination, which accounted for 41%–66% of total vaccination costs (Table 1). Excluding these time costs resulted in cost-effectiveness ratios approximately half of those reported in Table 5 and Figure 2. Using an alternative calculation for quality adjustments resulted in higher estimates of the projected number of QALYs gained through vaccination. For example, projected gains in QALYs for children not at high risk were 12%–37% higher than in the base case.

Two-way sensitivity analysis on influenza illness rate and vaccine effectiveness (IIV) resulted in changes in the cost-effectiveness ratio from a decrease in 11% for a season with a high influenza illness rate (35%) and high vaccine effectiveness (IIV) to an increase of more than a factor of 30 for seasons with a low influenza illness rate and low vaccine effectiveness (IIV). Combining a high influenza illness rate (35%) with low vaccine effectiveness (IIV) resulted in cost-effectiveness ratio ≈3 times base case results ($43,000/QALY) for children not at high risk ages 6–23 months. Two-way sensitivity analyses on influenza illness rate and total vaccination costs (IIV) had similar results, ranging from a decrease in 6% in the cost-effectiveness ratio to an increase 25 times as high as the base case for seasons with low influenza illness rate and high vaccination costs (IIV). Two-way sensitivity analyses for vaccination costs and effectiveness yielded a 20% lower cost-effectiveness ratio for low costs and high effectiveness of vaccination (IIV) to 7 times the base case for high costs and low vaccine effectiveness (IIV).

## Discussion

### Major Findings

We found that influenza vaccination of children, both those at high risk and those not at high risk, in all age groups would have health benefits that outweigh vaccine adverse events as measured by QALYs for both IIV and LAIV. For children not at high risk ages 6 months–4 years, we estimated that influenza vaccination with IIV would cost <$25,000 per QALY saved. In comparison, other routinely-used preventive interventions, such as pneumococcal conjugate vaccination, cost an average of $7,000/QALY for children <2 years (*22**,**25*); driver-side air bags cost $30,000/QALY (*26*), and costs of other vaccinations range from cost-savings to $150,000/QALY (*27**–**30*).

Live, attenuated influenza vaccine is currently approved for children >5 years of age who are not at high risk, but not for children <5 years or for children at high risk. At a price per dose <$20, its cost-effectiveness ratios are similar to those for IIV. This analysis likely presents a relatively conservative estimate of the potential benefits of LAIV, because we did not include its potentially greater effectiveness against antigenically drifted strains or likely higher effectiveness with 1 dose of vaccine in previously unvaccinated children <9 years (*4**,**6*).

The sensitivity of the results to the influenza illness attack rate (which varies from season to season and from community to community) and to vaccine effectiveness indicates that the cost-effectiveness of influenza vaccination can vary considerably from year to year. In seasons with a low influenza attack rate, the cost-effectiveness of vaccination with IIV would be dramatically higher than in the base case (Figure 3). The 2-way sensitivity analyses demonstrate even less favorable cost-effectiveness for a scenario that assumes a low influenza illness rate and low level of vaccine effectiveness. In addition, the sensitivity of these results to the total costs of vaccination highlights the potential for delivering vaccinations in settings that have lower costs and reduce the time required for vaccination.

### Comparisons with Previous Studies

Our study contributes valuable new information because it incorporates survey-based health state preferences for influenza-related illness and vaccine adverse events. These preferences, which are expressed as QALYs saved, are important for 2 reasons. First, we were able to evaluate the net health benefits of vaccination by subtracting the QALYs lost due to vaccine adverse events from the QALYs gained due to averted influenza cases. The results suggest that vaccination of all children is desirable from a health standpoint. Second, the outcome measure of dollars per QALY saved allows policymakers to compare the cost-effectiveness of influenza vaccination of children with other potential investments in preventive health services.

Authors of other economic analyses of influenza vaccination in children concluded that vaccination was more cost-effective than we found in our study (*8**,**9**,**31*). However, in these studies, the authors either did not separate children at high risk from those not at risk (*31*) or did not allow for sufficient variability in key variables (*8**,**9*). These studies assumed substantially higher influenza attack rates (*8**,**9*), higher levels of vaccine effectiveness (*8**,**9*), and lower total costs of vaccination (*8**–**10*), all of which would favor vaccination. However, we believe that it is more accurate to include variation in both incidence of influenza-related clinical illnesses and rates of influenza-related health outcomes. Neither our study nor the previous studies included potential benefits of herd immunity.

In a recent study that used cost-benefit analysis to evaluate the economics of influenza vaccination in children, Meltzer et al. arrived at conclusions similar to this analysis for many of the age/risk groups under consideration (*32*). Meltzer et al. found that annual vaccination of children not at high risk was unlikely to be cost-saving and that annual vaccination of children 6–23 months at high risk was likely to generate cost savings. For older children at high risk, they estimated median cost savings, but this analysis projects net costs of influenza vaccination for similar-age risk groups.

### Limitations

Some studies that used mathematical models have suggested substantial community herd immunity effects from vaccinating school-aged children (*33*). Although one study demonstrated herd immunity with vaccination rates of >80% among school-aged children during the 1968 pandemic (*34*), a recent study by Pisu et al. (*35*) reported that vaccinating 20%–25% of children <5 years of age in a Texan community did not generate any measurable herd immunity in persons <35 years. Additionally, no field studies have assessed the impact of pediatric vaccination on hospitalization and deaths in adults. Thus, we made the conservative decision to not include herd immunity effects in our analysis. If herd immunity effects had been included in our analysis, the findings would likely have been more favorable for vaccination. Future analyses should evaluate the cost-effectiveness of expanding routine influenza immunization under different assumptions for vaccine coverage rates and the costs of achieving these rates.

A recent randomized trial suggests that influenza vaccination has little, if any, effect on otitis media in children (*36*), while previous trials have found that influenza vaccination reduces otitis media (*6**,**37*). Our model assumes that only a small proportion of otitis media is preventable by influenza vaccination, and our findings are consistent with estimates of otitis media reduction from influenza vaccination reported in all of these studies. Our model is conservative in that it only includes the effect of reduced incidence of otitis media (or other complications) due to reduced incidence of influenza illness and does not consider any other benefits of vaccination, such as whether vaccinated patients with influenza illness may have a lower probability of experiencing otitis media (or other complications).

The time-tradeoff questions we used to elicit preferences for health states differ from that commonly used for adult illnesses because the loss of quality of life for both parent and child are explicitly included. In addition, parents were asked to include the value of productivity losses to paid or unpaid work for caring for a child with influenza in the time-tradeoff amount; therefore, productivity losses were included in the health state quality adjustments, whereas parent time costs for vaccination were included as dollar costs. As a result, the time-tradeoff amounts presented here are not directly comparable to utility values from generic utility instruments for measuring reductions in quality-of-life for chronic health states, such as the Health Utilities Index (*38*) or the EQ-5D (*39*). The sample sizes for the time-tradeoff studies were small.

Recent data show that some influenza-related deaths in children may occur outside the medical setting (*2*). Only deaths that occurred after an influenza-related hospitalization have been included in this analysis. However, even a 10-fold increase in influenza-related deaths did not appreciably change the cost-effectiveness results since the total number of deaths remains small.

Few data are available to guide assumptions on what proportion of children who experience mild systemic symptoms after vaccination, such as fever or respiratory symptoms, will see a physician. In the absence of reliable data, we selected an assumption that would be more likely to bias against vaccination rather than for and assumed it would be the same as the proportion of children who would visit a physician due to influenza illness. If the number of medically attended, vaccination-related adverse events were lower, the cost-effectiveness ratios would also be slightly lower, but cost-effectiveness results are not very sensitive to this parameter. We did not include any quality adjustment for vaccination itself aside from negative effects of vaccination-related adverse events. If vaccination itself were associated with a decrease in quality of life, cost-effectiveness ratios would be less favorable than in the current analysis. Previous analyses of other vaccinations, which included a quality adjustment for fever and fussiness following vaccination, were not sensitive to this parameter (*22**,**40*).

## Conclusions

Routine annual influenza vaccination using IIV for children age >2 years not at high risk is likely to result in net health benefits, but cost-effectiveness ratios are likely to be less favorable than for children ages 6–23 months and children of any age with a high-risk condition. Cost-effectiveness among children decreases with increasing age, although risk status is more important than age in determining the economic impact of annual influenza vaccination. Further work is needed to assess the potential impact of herd immunity on the cost-effectiveness of expanding influenza vaccine recommendations.

## Appendix

Health Benefits, Risks, and Cost-Effectiveness of Influenza Vaccination in Children

This appendix provides additional information on methods and data and is intended to supplement the corresponding article.

Equation for Calculating Cost-Effectiveness (CE) Ratios

Table A1

**Table A1 References**

1. Fox JP, Hall CE, Cooney MK, Foy HM. Influenza virus infections in Seattle families, 1975–1979: II. Pattern of infection in invaded households and relation of age and prior antibody to occurrence of infection and related illness. Am J Epidemiol. 1982;116:228–42.

2. Foy HM, Hall CE, Cooney MK, Allan ID, Fox JP. Influenza surveillance in the Pacific Northwest 1976-1980. Int J Epidemiol. 1983;12:353–6.

3. Glezen WP, Taber LH, Frank AL, Gruber WC, Piedra PA. Influenza virus infections in infants. Pediatr Infect Dis J. 1997;16:1065–8.doi:10.1097/00006454-199711000-00012

4. Hall CE, Cooney MK, Fox JP. The Seattle virus watch: IV. Comparative epidemiologic observations of infections with influenza A and B viruses, 1965-1969, in families with young children. Am J Epidemiol. 1973;98:365–80.

5. Monto AS, Sullivan KM. Acute respiratory illness in the community: frequency of illness and the agents involved. Epidemiol Infect. 1993;110:145–60.

6. Monto AS, Koolman JS. Longini Jr IJ. Tecumseh study of illness: XIII. Influenza infection and disease, 1976-1981. Am J Epidemiol. 1985;121:811–22.7. Neuzil KM, Zhu Y, Griffin MR, Edwards KM, Thompson JM, Tollefson SJ, et al. Burden of interpandemic influenza in children younger than 5 years: a 25-year prospective study. J Infect Dis. 2002;185:147–52.

8. Neuzil KM, Dupont WD, Wright PF, Edwards KM. Efficacy of inactivated and cold-adapted vaccines against inlfuenza A infection, 1985 to 1990: the pediatric experience. Pediatr Infect Dis J. 2001;20:733–40.

9. Sullivan KM, Monto AS. Longini Jr IJ. Estimates of the US health impact of influenza. Am J Public Health. 1993;83:1712–6.

10. Taber LH, Paredes A, Glezen WP, Couch RB. Infection with influenza A/Victoria virus in Houston families, 1976. J Hyg (Lond). 1981;86:303–13.

11. Neuzil KM, Mellen BG, Wright PF, Mitchel EF, Griffin MR. The effect of influenza on hospitalizations, outpatient visits, and courses of antibiotics in children. N Engl J Med. 2000;342:225–31.

12. Neuzil KM, Wright PF, Mitchel EF, Griffin MR. The burden of influenza illness in children with asthma and other chronic medical conditions. J Pediatr. 2000;137:856–64.

13. Belshe RB, Mendelman PM, Treanor J, King J, Gruber WC, Piedra PA, et al. The efficacy of live attenuated, cold-adapted, trivalent, intranasal influenzavirus vaccine in children. N Engl J Med. 1998;338:1405–12.

14. Heikkinen T, Ruuskanen O, Waris M, Ziegler T, Arola M, Halonen P. Influenza vaccination in the prevention of acute otitis media in children. Am J Dis Child. 1991;145:445–8.

15. Heikkinen T, Thint M, Chonmaitree T. Prevalence of various respiratory viruses in the middle ear during acute otitis media. N Engl J Med. 1999;340:260–4. doi:10.1056/NEJM199901283400402

16. Teele DW, Klein JO, Rosner B; Greater Boston Otitis Media Study Group. Epidemiology of otitis media during the first seven years of life in children in greater Boston: a prospective, cohort study. J Infect Dis. 1989;160:83–94. doi:10.1093/infdis/160.1.83

17. Izurieta HS, Thompson WW, Kramarz P, Shay DK, Davis RL, DeStefano F, et al. Influenza and the rates of hospitalization for respiratory disease among infants and young children. N Engl J Med. 2000;342:232–9. doi:10.1056/NEJM200001273420402

18. Thompson WW, Shay DK, Weintraub E, Brammer L, Cox N, Anderson LJ, et al. Mortality associated with influenza and respiratory syncytial virus in the United States. JAMA. 2003;289:179–86.

19. Nichol KL. The efficacy, effectiveness and cost-effectiveness of inactivated influenza virus vaccines. Vaccine. 2003;21:1769–75.

20. Belshe RB, Gruber WC, Mendelman PM, Cho I, Reisinger K, Block SL, et al. Efficacy of vaccination with live attenuated, cold-adapted, trivalent, intranasal influenza virus vaccine against a variant (A/Sydney) not contained in the vaccine. J Pediatr. 2000;136:168–75.

21. Drug topics redbook. Montvale (NJ): Medical Economics Co.; 2000.

22. Finkelstein JA, Stille CS, Nordin J, Davis R, Raebel MA, Roblin D, et al. Reduction in antibiotic use among US children, 1996-2000. Pediatrics. 2003;112:620–7.

23. Waitzman NJ, Scheffler RM, Romano PS. The cost of birth defects: estimates of the value of prevention. Lanham (MD): University Press of America, Inc.; 1984.

24. Clabaugh J. MedImmune slashes FluMist price, finds distributor. Washington Business Journal. 2004 Jun 15.

25. Consumer price index. US Department of Labor Statistics. 2004 [cited 2004 Aug 16]. Available from http://www.bls.gov/cpi/

26. American Academy of Pediatrics. Pediatric service utilization, fees and managed care arrangements: 2001 report based on 1999 data. Elk Grove Village (IL): The Academy; 2001. p. 1–11.

27. Prosser LA, Bridges CB, Uyeki TM, Rego VH, Ray GT, Meltzer MI, et al. Values for preventing influenza-related morbidity and vaccine adverse events in children. Health Qual Life Outcomes. In Press.

28. Prosser LA, Ray GT, O'Brien M, Kleinman K, Santoli J, Lieu TA. Preferences and willingness to pay for health states prevented by pneumococcal conjugate vaccine. Pediatrics. 2004;113:283–90.

Table A2

Table A3

## Appendix

**Table A1:** Expanded list of model inputs

Variable		Most likely estimate	Range for sensitivity analysis	Source	Type of distribution	Distribution parameter 1	Distribution parameter 2
Influenza illness attack rate (annual)				(1–10)			
	6–23 mo	0.157	0.02–0.35		β^1^	2.2	11.8
	2 y	0.155	0.02–0.35		Derived^2^		
	3–4 y	0.155	0.02–0.35		Derived		
	5–11 y	0.08	0.01–0.18		Derived		
	12–17 y	0.06	0.01–0.14		Derived		
Probability of an outpatient visit for child with influenza illness^3^				(5,11,12)4			
	6–23 mo	0.5	0.17–0.83		β	3.3	3.5
	2 y	0.47	0.15–0.81		β	3.29	3.71
	3–4 y	0.43	0.12–0.78		β	3.01	3.99
	5–11 y	0.28	0.11–0.5		β	5.6	14.4
	12–17 y	0.24	0.06–0.5		β	2.88	19.12
Probability of otitis media for a child with medically attended influenza illness				(13–16), expert panel			
	6–23 mo	0.63	0.33–0.8		β	6.3	3.7
	2 y	0.58	0.27–0.8		β	5.22	3.78
	3–4 y	0.39	0.17–0.6		β	6.24	9.76
	5–11 y	0.23	0.05–0.5		β	2.53	8.47
	12–17 y	0.15	0.01–0.4		β	1.5	8.5
Probability of nonhospitalized pneumonia or other outpatient complication for child with medically attended influenza illness^5^				(11,12); expert panel			
	6–23 mo	0.2	0.04–0.5		β	2.6	10.4
	2 y	0.15	0.02–0.4		β	1.95	11.05
	3–4 y	0.15	0.02–0.4		β	1.95	11.05
	5–11 y	0.11	0.02–0.3		β	2.2	17.8
	12–17 y	0.08	0.01–0.2		β	2.16	24.84
Hospitalizations for pneumonia or other respiratory conditions due to influenza per 10,000 children not at high risk^6^				(7,11,17); W. Thompson, pers. comm.)			
	6–23 mo	28.3	1.9–80.0		β	5.5	244.5
	2 y	17.1	0–56.8		β	3.4	246.6
	3–4 y	8.0	0–35.4		β	1.6	248.4
	5–11 y	3.1	0–16.0		β	7.95	1,492.1
	12–17 y	3.1	0–14.9		β	10.5	1,489.5
Probability of long-term sequelae after influenza-related hospitalization^2^		0.01	0.001–0.03	Expert panel	β	1.3	11.7
Probability of death during influenza-related hospitalization		0.0009	0–0.002	(18)4	β	1.7	18.3
Vaccine effectiveness in preventing influenza illness9							
	IIV	0.69	0.4–0.9	(19)4	β	7.59	3.41
	LAIV	0.838	0.6–0.96	(20)4	β	16.76	3.24
Probability of medically attended vaccination-related adverse events							
Injection site reaction							
	6–23 mo	0.008	0.002–0.017	(8)	β	4.0	46.0
	2 y	0.003			Derived^10^		
	3–4 y	0.002			Derived		
	5–11 y	0.001			Derived		
	12–17 y	0.0003			Derived		
Systemic reaction (fever)11							
	6–23 mo	0.013	0.001–0.025	(20)	β	5.2	194.8
	2 y	0.011			Derived		
	3–4 y	0.009			Derived		
	5–11 y	0.004			Derived		
	12–17 y	0.003			Derived		
	Anaphylaxis	0.00000025	0–0.000001	Expert panel	β^12^	0.5	19.5
	Guillain-Barré syndrome	0.000001	0–0.00001	Expert panel	Triangular	0.000001 (most likely)	0 (min), 0.000002 (max)
Influenza-related costs							
	OTC medications^13^	$3		(21,22); J. Finkelstein, pers. comm.; expert panel			
	Physician visit for uncomplicated influenza^14^	$27	$0–$180	Marketscan database15	Lognormal^16^	32	27
Physician visit for otitis media							
	6–3 mo	$78	$23–$197	Marketscan database^17^	Lognormal	98	78
	2–4 y	$83	$23–$200	Marketscan database^17^	Lognormal	100	83
	5–17 y	$94	$31–$245	Marketscan database^17^	Lognormal	117	94
Physician visit for nonhospitalized pneumonia							
	6–23 mo	$179	$62–$715	Marketscan database^17^	Lognormal	252	179
	2–4 y	$88	$27–$333	Marketscan database^17^	Lognormal	130	88
	5–17 y	$109	$34–$503	Marketscan database^17^	Lognormal	187	109
Hospitalization^18^							
	6–23 mo	$4,306	$1,307–$34,473	Marketscan database^17^	Lognormal	13194	4306
	3–4 y	$4,180	$1,292–$32,030	Marketscan database^17^	Lognormal	10000	4180
	5–17 y	$5,135	$1,373–$42,990	Marketscan database^17^	Lognormal	14956	5135
Long-term sequelae following influenza-related hospitalization^19^		$625,000	$0–$1,000,000	(23)			
Vaccination costs							
	Per dose, IIV^20^ (children <3 y)	$9.56^21^	1×–4× base case	(21)			
	Per dose, IIV (children >3 y)	$6.86^21^	1×–4× base case	(21)			
	Per dose, LAIV^20^	$12.89^22^	$10–$25	(24,25)			
	Administration (0–2 visits)^23^	$25	$10–$40	(26)			
	Parent time costs^24^	$32	$0–$62	(27), expert panel			
	Total vaccination costs		$30–$110				
	6–23 mo	$79					
	2 y	$66					
	3–4 y	$59					
	5–11 y	$49					
	12–17 y	$49					
Vaccination-related adverse events							
	Physician visit for injection site reaction^25^	$61	$30–$-683	Marketscan database^26^	Lognormal^16^	202	61
	Anaphylaxis^27^	$2,699	$52–$13,754	Marketscan database^28^	Lognormal^16^	4527	2699
	Guillain-Barré syndrome^29^	$23,359	$6,663–$78,912	Marketscan database^28^	Lognormal^16^	32196	23359
Quality adjustments^30,31^ (disutility associated with an event)							
	Episode of influenza	0.005	0.002–0.009	(*27*)	β	7.35	1492.65
	Otitis media	0.042	0.023–0.065	(*28*)	β	14.56	335.44
	Nonhospitalized complications (pneumonia)	0.046	0.027–0.071	(28)	β	16.21	333.8
	Hospitalization, pneumonia	0.076	0.054–0.100	(28)	β	37.85	462.15
	Anaphylaxis	0.02	0.006–0.041	(27)	β	4.53	225.47
	Guillain-Barré syndrome	0.141	0.092–0.199	(27)	β	22.53	137.47

IIV, inactivated influenza vaccine; LAIV, live, attenuated influenza vaccine; OTC, over the counter. ^1^Distributions for transition probabilities were assigned using most likely values and ranges identified in the literature and/or expert panel. For these parameters, primary data were not available and beta distributions were assigned to match the values identified in the table. ^2^Distributions for age groups other than 6–23 mo are based on the 6- to 23-mo distribution multiplied by the ratio of the most likely estimates for the age group in question to children 6–23 mo (e.g., the distribution for 2 y is calculated by multiplying the distribution for 6–23 mo by 0.155/0.157). ^3^Estimates for healthy children are shown in Table. Probabilities are estimated to be twice as high for children at high risk for influenza-related complications. ^4^Range for sensitivity analysis determined by expert opinion. ^5^Estimates for healthy children shown in Table. Probabilities are estimated to be up to 5 times as high for children at high risk for influenza-related complications. Base case estimates for children at high risk are 1.6 times as high as for healthy children. ^6^Children at high risk are estimated to be hospitalized at 3–6 times the rate of healthy children. ^7^Probability from distribution divided by 10. ^8^Probability from distribution divided by 100. ^9^Assumes vaccine is poorly matched with circulating virus 1 in 10 years (i.e., vaccine effectiveness is assumed to be 0 in years with a poor match). ^10^Distributions for age groups other than 6–23 mo are based on the 6- to 23-mo distribution multiplied by the ratio of the most likely estimates for the age group in question to children ages 6–23 mo (e.g., the distribution for 2 years is calculated by multiplying the distribution for 6–23 mo by 0.003/0.008). ^11^Definitions and follow-up for incidence of fever following vaccination vary by study. Rates are 2× higher for high-risk subgroups. ^12^Probability from distribution divided by 100,000. ^13^Vary by age, calculated by costing out recommended dose of acetaminophen for average weight in each age group. ^14^Only a proportion of children with influenza illness are assumed to make a physician visit. ICD-9 codes: 487 and 487.0. ^15^1993–1997 Marketscan database, The Medstat Group, Ann Arbor, MI, USA. ^16^Lognormal distributions are approximated using the mean and median in Treeage. In this table, parameter 1 is the mean and parameter 2 is the median for each distribution. ^17^2001-2003 Marketscan database, The Medstat Group, Ann Arbor, MI. ^18^ICD-9 codes: 460-466, 471-474, 477, 478, 480-483, 490-496, 506-508, 510, 511, 514, 518, 519. 2001-2003 Marketscan database. ^19^Includes costs of lifetime care and special education. ^20^Assumed 2 doses will be required for children <5 years receiving their first influenza vaccination. ^21^Vaccine dose costs are based on 2004 CDC negotiated prices. Cost for children <3 years assumes thimerosal-free vaccine is used. ^22^Based on 2004 CDC negotiated price. ^23^Common Procedural Terminology (CPT) codes: 99211, 90471. Physician costs for vaccine administration at existing visit is $10.37 (90471); $19.95 for vaccine administration requiring a separate visit (99211). ^24^Each physician visit is assumed to take 2 hours of parent time valued at an average hourly wage rate of $15.54. ^25^5- minute visit, CPT code 99211. ^26^ 2001–2003 Marketscan database. ^27^ICD-9 codes: 999.4, 995.0, 995.6x. ^28^2001-2003 Marketscan database. ^29^ICD-9 code: 357.0. ^30^Quality adjustments are included in the model as a one-time decrement in utility for each temporary health state. For example, an episode of influenza results in a one-time loss of 0.005 quality-adjusted life years (QALYs). Utility losses were calculated by dividing the discounted time-traded off by the respondent’s discounted life expectancy. ^31^Average life span used to calculate total QALYs lost due to life-long sequelae and death was 77.9–78.2 y, depending on child’s current age. See Table A1 References in Appendix.

**Table A2:** No. additional physician visits required to administer required doses, by age group

Age group	No extra visits required	One extra visit required	Two extra visits required
6–23 mo	0.27	0.39	0.34
2 y	0.26	0.57	0.17
3–4 y	0.26	0.64	0.10
5–11 y	0.25	0.75	–
12–17 y	0.25	0.75	–

**Table A3:** Health benefits, risks, and costs of influenza vaccination of varying age and risk groups, means (95% CI)*†‡

		Per 1,000 children							
		Cost of vaccination program§	Savings from influenza disease averted	Net costs	Influenza events averted (all)	Influenza hospitalizations averted	Deaths averted	Vaccine adverse events incurred (medically attended)¶	QALYs gained (95% CI^32^)
Healthy		Using inactivated influenza vaccine							
	6–23 mo	$79,000	$42,000	37,000 (–119,000 to 98,000)	108 (16–276)	2 (0.2–6)	0.002 (0–0.007)	21 (8–47)	3.0 (0.4–9.0)
	2 y	$66,000	$23,000	43,000 (–40,000 to 83,000)	107 (15–276)	1.2 (0.1–4.2)	0.001 (0–0.005)	14 (5–30)	2.4 (0.3–7.3)
	3–4 y	$59,000	$12,000	47,000 (2,000–78,000)	107 (15–276)	0.6 (0; 2.3)	0.0005 (0–0.0025)	10 (3–24)	1.7 (0.2–5.2)
	5–11 y	$49,000	$5,000	44,000 (21,000–68,000)	55 (8–142)	0.2 (0–0.7)	0.0002 (0–0.0008)	*5 (2–11)*	0.6 (0.1–1.7)
	12–17 y	$49,000	$5,000	44,000 (22,000–68,000)	41 (6–104)	0.2 (0–0.6)	0.0002 (0–0.0008)	*3 (1–8)*	0.4 (0–1.1)
High risk:		Using inactivated influenza vaccine							
	6–23 mo	$79,000	$153,000	–74,000) (–552,000 to 83,000)	108 (16–276)	5.5 (0.5–6.5)	0.005 (0–0.020)	32 (11–56)	7.2 (0.8–23.2)
	2 y	$66,000	$88,000	–22,000) (–292,000 to 72,000)	107 (15–276)	3.5 (0.2–11.4)	0.003 (0–0.013)	25 (7–44)	5.4 (0.6–17.2)
	3–4 y	$59,000	$57,000	2,000 (–212,000 to 70,000)	107 (15–276)	2.2 (0.1–9.1)	0.002 (0–0.010)	19 (5–37)	4.0 (0.4–13.1)
	5–11 y	$49,000	$37,000	12,000 (–125,000 to 59,000)	55 (8–142)	1.3 (0.1–3.9)	0.001 (0–0.005)	9 (3–24)	1.6 (0.2–5.6)
	12–17 y	$49,000	$36,000	13,000 (–120,000 to 59,000)	41 (*6; 104*)	1.3 (0.1–3.9)	0.001 (0–0.005)	*6 (1–15)*	1.3 (0.1–4.5)
Healthy		Using live attenuated influenza vaccine							
	6–23 mo	$99,000	$63,000	*32,000 (–155,000 to 99,000)*	*132 (20–319)*	*2.4 (0.3–7.2)*	*0.002 (0–0.009)*	*13 (3–32)*	*3.7 (0.5–10.5)*
	2 y	$80,000	$35,000	*42,000 (–59,000 to 85,000)*	*130 (20–322)*	*1.4 (0.1–4.9)*	*0.001 (0; 0.005)*	*11 (2–26)*	*2.9 (0.4–8.5)*
	3–4 y	$74,000	$21,000	*50,000 (–3,000 to 83,000)*	*130 (20–322)*	*0.7 (0–2.7)*	*0.0006 (0–0.0029)*	*9 (2–23)*	*2.1 (0.3–6.1)*
	5–11 y	$61,000	$11,000	48,000 (22,000–73,000)	67 (10–166)	0.3 (0–0.8)	0.0002 (0–0.0010)	*4 (1–10)*	0.7 (0.1–1.9)
	12–17 y	$61,000	$10,000	49,000 (23,000–73,000)	50 (8–120)	0.3 (0–0.7)	0.00002 (0–0.0010)	3 (0–7)	0.5 (0.1–1.3)

*CI, confidence interval; QALYS, quality-adjusted life years; italics indicate that live, attenuated influenza vaccine is not licensed for children <5 y. †Bootstrapped. ‡Figures may not sum due to rounding. §Includes time costs associated with vaccination. ¶Includes injection site reactions, systemic reactions, anaphylaxis, and Guillain-Barré syndrome.
